# Screening for New Surface Anchoring Domains for *Lactococcus lactis*

**DOI:** 10.3389/fmicb.2019.01879

**Published:** 2019-08-13

**Authors:** Tina Vida Plavec, Borut Štrukelj, Aleš Berlec

**Affiliations:** ^1^Department of Biotechnology, Jožef Stefan Institute, Ljubljana, Slovenia; ^2^Faculty of Pharmacy, University of Ljubljana, Ljubljana, Slovenia

**Keywords:** surface display, *Lactococcus lactis*, anchor, phage AM12, ChW, LPXTG, endolysin

## Abstract

The display of recombinant proteins on bacterial surfaces is a developing research area with a wide range of potential biotechnological applications. The lactic acid bacterium *Lactococcus lactis* is an attractive host for such surface display, and a promising vector for *in vivo* delivery of bioactive proteins. Surface-displayed recombinant proteins are usually anchored to the bacterial cell wall through anchoring domains. Here, we investigated alternatives to the commonly applied lactococcal lysine motif (LysM)-containing surface anchoring domain, the C-terminus of AcmA (cAcmA). We screened 15 anchoring domains of lactococcal or phage origins that belong to the Pfam categories LPXTG, LysM, CW_1, Cpl-7, WxL, SH3, and ChW, which can provide non-covalent or covalent binding to the cell wall. LPXTG, LysM, the duplicated CW_1 and SH3 domains promoted significant surface display of two model proteins, B domain and DARPin I07, although the display achieved was lower than that for the reference anchoring domain, cAcmA. On the other hand, the ChW-containing anchoring domain of the lactococcal phage AM12 endolysin (cAM12) demonstrated surface display comparable to that of cAcmA. The anchoring ability of cAM12 was confirmed by enabling non-covalent heterologous anchoring of the B domain on wild-type bacteria, as well as anchoring of CXCL8-binding evasin-3, which provided potential therapeutic applicability; both were displayed to an extent comparable to that of cAcmA. We have thereby demonstrated the effective use of different protein anchoring domains in *L. lactis*, with ChW-containing cAM12 the most promising alternative to the established approaches for surface display on *L. lactis*.

## Introduction

Bacteria with surface-displayed recombinant proteins are useful for numerous biotechnological applications. Protein-displaying bacteria can act as bioadsorbents, biosensors, biocatalysts, and oral vaccines. They can be exploited in antibody production and peptide screening (Georgiou et al., 1997; Stahl and Uhlen, 1997; Lee et al., 2003). Lactic acid bacteria (LAB) are valuable host organisms in biotechnology, due to their safety profile (i.e., “generally recognized as safe” status), long-term use in food, industrial applicability, and potential beneficial influence on health (i.e., probiotic properties). They are attractive for therapeutic applications due to their intrinsic health benefits (de Vrese and Schrezenmeir, 2008). What is more, their therapeutic potential can be increased by genetic engineering (Plavec and Berlec, 2019).

The display of heterologous proteins on the surface of LAB has already been exploited for therapeutic applications. This was used to prepare mucosal vaccines (van Roosmalen et al., 2006; Berlec et al., 2013; Van Braeckel-Budimir et al., 2013; de Azevedo et al., 2015; Michon et al., 2016) and to display binding molecules directed against pro-inflammatory cytokines, including tumor necrosis factor-α, interleukin (IL)-17, and IL-23 (Ravnikar et al., 2010; Berlec et al., 2017; Kosler et al., 2017), and chemokines (Skrlec et al., 2017), for the treatment of inflammatory bowel disease. Furthermore, *Lactococcus lactis* with β-galactosidase displayed on its cell surface was successfully constructed as a candidate for management of lactose intolerance (Yin et al., 2018). Further, for diabetes, a single-chain insulin analog (SCI-59) was secreted from *L. lactis* NZ3900 and displayed on non-viable bacteria (Mao et al., 2017). *L. lactis* was also tested for protection against HPV-16–induced tumors by the display on its surface of both the HPV-16-E7 antigen and fibronectin binding protein A from *Staphylococcus aureus.* This modified *L. lactis* elicited a more efficient systemic immune response compared to its wild-type counterpart (Almeida et al., 2016).

Lactic acid bacteria can also be used as biocatalysts through the display of different enzymes on their surface. For the production of chito- and manno-oligosaccharides, β-mannanase and chitosanase were successfully displayed on the surface of *Lactobacillus plantarum*, with confirmation of this biocatalytic action (Nguyen et al., 2016). Also for *L. plantarum*, self-assembling cellulosomal complexes with their catalytic subunits were displayed on the surface, and these showed efficient degradation of wheat straw (Stern et al., 2018). Similarly, chimeric protein scaffolds of type 1 and type 2 cohesins have been anchored to *L. lactis*, and the positioning of the enzymes on these scaffolds was shown to affect the catalytic profiles of the complexes (Wieczorek and Martin, 2012).

A protein to be displayed is usually fused to an anchoring domain (Desvaux et al., 2018). Different types of surface anchoring domains have been described for LAB, which include LPXTG-type domains (Dieye et al., 2001, 2010), lipoprotein anchors (Zadravec et al., 2014), surface layer proteins (Hu et al., 2011), and lysine motif (LysM) domains (Visweswaran et al., 2014). The most frequently applied surface anchoring domains in the prototype LAB *L. lactis* are: (i) the C-terminal domain of endogenous AcmA (cAcmA), which provides non-covalent anchoring through three peptidoglycan-binding LysM repeats (Steen et al., 2005; Andre et al., 2008; Okano et al., 2008); and (ii) the LPXTG sequence of the M6 protein of *Streptococcus pyogenes*, which provides covalent anchoring (Dieye et al., 2001; Kyla-Nikkila et al., 2010). Despite these available options, alternative surface display approaches are being sought, to allow diverse mechanisms of anchoring, as well as anchoring to different surface regions to achieve the simultaneous binding of different proteins.

The search for alternative surface anchoring domains is mitigated by the prediction of numerous anchoring domains in the Pfam database (El-Gebali et al., 2019). Non-covalent anchoring domains can have different lengths (e.g., 20–200 amino-acid residues) and can be repeated up to 64 times in a single protein, although up to 12 repeats is more common. They can be located at the N-terminus or C-terminus of a protein, or centrally (Zadravec et al., 2015a). Their genomic occurrence for the genus *Lactobacillus* was recently reviewed (Zadravec et al., 2015a); however, to date, most of the anchoring domains have not been characterized biochemically. Apart from native lactococcal proteins, proteins from lactococcal phages might represent a source of new non-covalent surface anchors (Oliveira et al., 2013). Host cell lysis and release of phages is achieved by the actions of endolysins, which are proteins that hydrolyze the cell wall. They usually include an N-terminal hydrolytic domain and a C-terminal cell-wall binding domain. The LysM anchor of endolysin of the *Lactobacillus fermentum* bacteriophage ΦPYB5 has been successfully displayed on the surface of several bacteria, including *L. lactis* (Hu et al., 2010).

The goal of the present study was to test some new anchoring domains for surface display on LAB *L. lactis*, and to compare them to the already known anchoring domains, considering in particular the most commonly used: cAcmA. This was achieved by preparing genetic constructs that comprised a reporter protein and a surface anchoring domain, of either lactococcal or phage origin. Two covalent binding LPXTG domains and 13 non-covalent anchoring domains were tested. Efficient surface display was achieved with several of these anchoring domains, of which at least one, cAM12, provided surface display comparable to that of cAcmA. cAM12 therefore represents an attractive new domain for anchoring recombinant proteins of interest to the surface of *L. lactis.*

## Materials and Methods

### Bacterial Strains, Media, and Growth Conditions

The bacterial strains used in this study are listed in Supplementary Table S1. *Escherichia coli* strain DH5α was grown under aeration at 37°C, in lysogeny broth medium supplemented with ampicillin (100 μg/mL). *L. lactis* NZ9000 was grown without aeration in M-17 medium (Merck) supplemented with 0.5% glucose (GM-17) and chloramphenicol (10 μg/mL), at 30°C.

### Bioinformatic Search for Anchoring Domains for *L. lactis*

Covalent LPXTG anchoring domains (Pfam number, PF00746) in the genome of *L. lactis* MG1363 were identified using LocateP (Zhou et al., 2008). The Pfam domains^1^ associated with non-covalent surface anchoring in bacteria were defined (Supplementary Material S1). The IMG/M tool^2^ was used to identify proteins of *L. lactis* that contain at least one of these domains. The protein sequences of endolysins of lactococcal phages were identified in GenBank, and the presence of anchoring domains checked using Pfam searches.

### Molecular Cloning

Plasmid DNA was isolated using NucleoSpin Plasmid (Macherey and Nagel, Düren, Germany), with an additional lysozyme treatment step for *L. lactis*. Lactococci were transformed with electroporation using the Gene Pulser II apparatus (Biorad, Hercules, CA, United States) according to the manufacturer instructions (MoBiTec GmbH, Goettingen, Germany). Nucleotide sequencing was performed by GATC Biotech (Konstanz, Germany).

The genes for the lactococcal anchoring domains (*2lysm*, *3lysm*, *cw*, *cpl*, *wxl1*, *wxl3*, *slpxtg*, and *llpxtg*) were amplified from the lactococcal genome by colony PCR, using the primers given in Supplementary Table S1, and cloned into the plasmid pGEM-T Easy. The genes were transferred to the plasmid pSDBA3b (Skrlec et al., 2017; pNZ8148 containing fusion of Usp45 secretion signal, B domain gene and cAcmA gene) via *Eco*RI/*Xba*I restriction enzyme recognition sites, to yield the following plasmids: pSD-2LysM, pSD-3LysM, pSD-CW, pSD-Cpl, pSD-WxL1, pSD-WxL3, pSD-sLPXTG, and pSD-lLPXTG (Supplementary Table S1). Reporter protein B domain is one of five antibody-binding domains of staphylococcal protein A (Moks et al., 1986; Ravnikar et al., 2010).

An alternative variant of *cw* (*cw-spe*) was amplified by PCR using the primers CW_1-F-Eco and CW_1-R-Spe, then digested with *Spe*I and ligated with *Xba*I-digested *cw* into the duplicated *cw* gene (*2cw*), using isocaudameric ligation. *2cw* was further amplified by PCR using the primers CW_1-F-Eco and CW_1-R-TAA-Xba, and then cloned into plasmid pSDBA3b via the *Eco*RI/*Xba*I restriction sites, to yield the plasmid pSD-2CW. The gene for pSD-2Cpl was designed likewise, using the primers Cpl-7-F-Eco, Cpl-7-R-Spe, and Cpl-7-R-TAA-Xba.

The genes for the lactococcal anchoring domains of phage origin (*am7*, *am12*, *sk1*, *1358*) were designed and synthesized *de novo* as gBlocks by Integrated DNA Technologies (Leuven, Belgium), then amplified by PCR using the primer pairs given in Supplementary Table S1, and cloned into pGEM-T Easy. The genes were then transferred to the plasmid pSDBA3b via the *Eco*RI/*Xba*I restriction sites, to yield the following plasmids: pSD-AM7, pSD-AM12, pSD-SK1, and pSD-1358 (Supplementary Table S1).

The genes for the lactococcal and phage anchoring domains in fusion with designed ankyrin repeat protein (DARPin) I07 (that binds the Fc domain of human IgGs (Steiner et al., 2008; Skrlec et al., 2015; Zadravec et al., 2015b) and belongs to the group of DARPins, which are small non-immunoglobulin protein scaffolds that can be selected against various targets) were prepared by replacing the B domain gene with the DARPin I07 gene (from plasmid pMA-T-I07) (Zadravec et al., 2015b), for the following plasmids: pSD-2LysM, pSD-3LysM, pSD-2CW, pSD-WxL3, pSD-AM7, pSD-AM12, and pSD-1358. This was achieved via the *Eco*RI/*Bam*HI restriction sites, yielding the following plasmids: pSD_I07, pDARP-2LysM, pDARP-3LysM, pDARP-2CW, pDARP-WxL3, pDARP-AM7, pDARP-AM12, and pDARP-1358 (Supplementary Table S1).

### Expression of Fusion Proteins in *L. lactis*

Overnight cultures of *L. lactis* harboring pSDBA3b, pSD-2LysM, pSD-3LysM, pSD-CW, pSD-Cpl, pSD-2CW, pSD-2Cpl, pSD-WxL1, pSD-WxL3, pSD-AM7, pSD-AM12, pSD-SK1, pSD-1358, pSD_I07, pDARP-2LysM, pDARP-3LysM, pDARP-2CW, pDARP-WxL3, pDARP-AM7, pDARP-AM12, pDARP-1358, pSD-sLPXTG, or pSD-lLPXTG were diluted (1:100) in 10 mL fresh GM-17 medium and grown to optical density OD_600_ = 0.8–1.0. Fusion protein expression was induced with 25 ng/mL nisin (Fluka AG, Buchs, Switzerland), for 3 h at 30°C. After this incubation, 1 mL of the cultures was stored at 4°C for flow cytometry analysis. The remaining cell culture was centrifuged at 5000 × *g* for 10 min. The cell pellets were resuspended in 400 μL phosphate-buffered saline (PBS; pH 7.4) and stored at −20°C for SDS-PAGE analysis. For the enzyme-linked immunosorbent assay (ELISA), the cell pellets were resuspended to optical density OD_600_ = 2.0 or OD_600_ = 6.0, and stored at 4°C. For testing the binding of the B domain–cAcmA and B domain–cAM12 fusion proteins (encoded by pSDBA3b and pSD-AM12, respectively) to non-recombinant bacteria, after centrifugation, the supernatant of the bacterial culture was decanted, filtered through 0.22-μm-pore-size filters (Millex-GV, Merck, Darmstadt, Germany) and stored at 4°C overnight.

### SDS-PAGE and Western Blotting

SDS-PAGE was performed with a Mini-Protean II apparatus (Bio-Rad, Hercules, CA, United States). Samples were thawed in an ice bath, briefly sonicated (UPS200S sonicator; Hielscher, Teltow, Germany), mixed with 2 × Laemmli sample buffer (4% SDS, 20% glycerol, 0.005% bromphenol blue and 0.125 M Tris HCl, pH 6.8), and dithiothreitol, and denatured by heating to 100°C before loading. The Page Ruler Plus (Fermentas, St. Leon-Rot, Germany) pre-stained standards were used for molecular weight comparisons. The proteins were stained with Coomassie brilliant blue or transferred to polyvinylidene fluoride membranes (Immobilon-P, Millipore) or nitrocellulose membranes (GE Healthcare Life Sciences, Marlborough, MA, United States), using either wet transfer at 100 V for 90 min, or semi-dry transfer with a protocol for 1.5-mm gels (Trans-Blot Turbo Blotting System; BioRad, CA, United States). The membranes were then blocked in 5% non-fat dried milk in TBS with 0.05% Tween-20 (TBST; 50 mM Tris–HCl, 150 mM NaCl, 0.05% Tween 20, pH 7.5), and for the domains in fusion with the B domain, incubated overnight at 4°C with a goat anti-protein A antibody (1:2500; Abcam) in 5% non-fat dried milk in TBST. Following three washes with TBST, the membranes were incubated for 2 h with a horse-radish peroxidase (HRP)-conjugated secondary donkey anti-goat IgG (1:5000; Jackson ImmunoResearch) in 5% non-fat dried milk in TBST. After three further washes with TBST, the membranes were incubated with the Lumi-Light chemiluminescent reagent (Roche). Images were acquired using a ChemiDoc MP imaging system (BioRad). To detect domains in fusion with DARPin I07, the membranes were incubated with fluorescein isothiocyanate (FITC)-conjugated human IgG (1:1000; Jackson ImmunoResearch, West Grove, PA, United States), and after washing with 0.05% TBST, the fluorescence was detected with a ChemiDoc MP imaging system (Bio-Rad), using blue excitation (488 nm).

### Flow Cytometry

For flow cytometry, 20 μL of cell cultures in the stationary phase was added to 500 μL Tris–buffered saline (TBS; 50 mM Tris–HCl, 150 mM NaCl, pH 7.5) and centrifuged at 5000 × *g* for 5 min at 4°C. To detect anchoring domains fused with the B domain, the pellets were resuspended in 500 μL TBS containing a goat anti-protein A antibody (1:2500). After 2 h of incubation at room temperature with constant shaking at 100 rpm, the cells were washed three times with 200 μL TBS with 0.01% Tween-20 (0.1% TBST), and resuspended in 500 μL TBS containing an Alexa Fluor 488 donkey anti-goat antibody (1:2500). After 2 h of incubation at room temperature with constant shaking at 100 rpm, the cells were washed three times with 200 μL 0.1% TBST and finally resuspended in 500 μL TBS. To detect the anchoring domains fused with DARPin I07, the pellets were resuspended in 500 μL TBS containing a FITC-conjugated primary human IgG antibody (1:250). After 2 h of incubation at room temperature with constant shaking at 100 rpm, the cells were washed three times with 200 μL 0.1% TBST, and finally resuspended in 500 μL TBS. The samples were analyzed using a flow cytometer (FACS Calibur; Becton Dickinson, Franklin Lakes, NJ, United States) at excitation of 488 nm and emission of 530 nm in the FL1 channel. The geometric mean fluorescence intensity (MFI) of at least 20,000 bacterial cells in the appropriate gate was measured. The means of at least three independent experiments were considered.

### Subcellular Fractionation of Recombinant *L. lactis* Containing Plasmids pSDBA3b and pSD-AM12

Cell fractions were prepared as described before (Visweswaran et al., 2017). 25 mL of overnight cultures of *L. lactis* expressing B domain–cAcmA and B domain–cAM12 fusion proteins were centrifuged. The cell pellet was resuspended in 1 mL of 50 mM sodium phosphate buffer (pH 6.5) containing 100 mM NaCl, 550 mM sucrose, 5 mg/mL lysozyme and 50 U of mutanolysin, and incubated for 1 h at 37°C. The cell wall fraction was collected after centrifugation of the cell suspension at 5000 × *g* for 15 min. The remaining cell pellet (protoplasts) were resuspended in 1 mL of 50 mM sodium phosphate buffer (pH 6.5) containing 100 mM NaCl and subjected to sonication (six pulses of 15 s spaced 30 s apart on ice) with a UPS200S sonicator (Hielscher, Teltow, Germany). Unbroken cells were removed by centrifugation (5000 × *g* for 15 min at 4°C) and the supernatant was centrifuged at 30,000 × *g* for 30 min. After centrifugation, the supernatant (cytoplasmic fraction) was collected and the pellet (membrane fraction) was resuspended in 1 mL of denaturation buffer containing 2% dithiothreitol, 15% sucrose, and 3.8% sodium dodecyl sulphate (SDS) (all w/v). Separated fractions were analyzed with SDS-PAGE and Western blot as described above for B domain fusion proteins.

### Binding of the B Domain – cAcmA and B Domain – cAM12 Fusion Proteins to Non-recombinant *L. lactis*

Cell cultures of *L. lactis* were grown to an optical density OD_600_ = 2.0 to 3.0 (late exponential phase). For flow cytometry, 20 μL of the cell cultures was added to 500 μL TBS and centrifuged at 5000 × *g* for 5 min at 4°C. This was followed by resuspension of the pellets in 500 μL B domain–cAcmA or B domain–cAM12 fusion-protein-containing conditioned media, which were obtained by culturing *L. lactis* harboring pSDBA3b or pSD-AM12, respectively. The producer cells were then removed (see section “Expression of Fusion Proteins in *L. lactis*”). Suspensions were incubated for 2 h at room temperature with constant vigorous shaking. After the incubation, the cells were washed once with 500 μL TBS and stained as described above for flow cytometry. The control samples were stained without prior binding of the B domain–cAcmA and B domain–cAM12 fusion proteins to the bacterial surface.

### Chemokine Binding by Evasin-3–Displaying *L. lactis*

Different volumes of *L. lactis* cultures expressing evasin-3-cAcmA and evasin-3-cAM12 fusion proteins, or non-recombinant *L. lactis* coated with evasin-3-cAcmA and B domain-cAM12 fusion proteins, were centrifuged at 5000 × *g* for 5 min at 4°C, washed twice with 500 μL PBS, and finally resuspended in 200 μL PBS containing the CXCL8 standard [from human IL-8 (CXCL8) ELISA development kits (HRP); Mabtech, Nacka Strand, Sweden] and incubated for 2 h at room temperature with gentle shaking. After the incubation, the cells were removed by centrifugation at 5000 × *g* for 10 min at 4°C, and 100 μL of the supernatants was collected for determination of the chemokine concentration using ELISA kits. The standard curve (range, 4–400 pg/mL) was prepared according to the manufacturer instructions. Nunc Maxisorp 96-well plates were coated with the recommended concentrations of the chemokine binding antibodies overnight at 4°C. Then 100 μL of the samples was added, with an incubation for 2 h at room temperature. The wells were washed five times with 200 μL PBS containing 0.05% Tween-20 (wash buffer). Then, 100 μL biotinylated monoclonal antibodies against the chemokine was added at the recommended concentration, and incubated for 1 h at room temperature. The wells were washed five times with 200 μL wash buffer, with the addition of 100 μL streptavidin-HRP (1:1000). After a 1-h incubation at room temperature, the plates were washed five times again with wash buffer, and 100 μL 3,3′,5,5′-tetramethylbenzidine substrate (TMB; Sigma Aldrich) was added. The TMB substrate reaction was stopped after 15 min by addition of 50 μL 2 M sulfuric acid. The absorbance was read at 450 nm using a microplate reader (Infinite M1000; Tecan), with wavelength correction at 650 nm.

### Statistical Analyses

Statistical analyses were performed using the GraphPad Prism 5.0 software. Student’s *t* tests were used to define the significances of the differences between the B domain-displaying bacteria and the respective control, and the DARPin I07-displaying bacteria and the respective control.

## Results

### Surface Display With Covalent Anchoring Domains sLPXTG and lLPXTG

**FIGURE 1:**
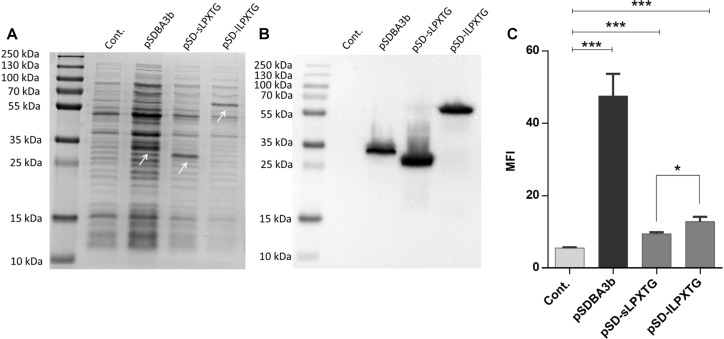
Coomassie staining **(A)** and Western blotting **(B)** of lysates of *L. lactis* cells expressing B domain in fusion with the Usp45 secretion signal and anchoring domains cAcmA (pSDBA3b), sLPXTG (pSD-sLPXTG), and lLPXTG (pSD-lLPXTG). B domain-anchoring domain fusion proteins are indicated with arrows. Flow cytometry **(C)** of *L. lactis* cells expressing the Usp45 secretion signal – B domain-anchoring domain fusion proteins, detected with an Alexa 488 labeled donkey anti-goat antibody. Cont., control, containing empty plasmid pNZ8148; MFI, mean fluorescence intensity. Error bars indicate standard deviations. ^∗^*p* < 0.05; ^∗∗∗^*p* < 0.001; as indicated (Student’s *t*-tests).

Ten LPXTG-containing proteins were identified in *L. lactis* MG1363 (parent of *L. lactis* NZ9000) genome using LocateP (Zhou et al., 2008). The LPXTG-containing C-terminal parts of the CluA protein [(Godon et al., 1994); accession number CAL97984.1] of two different lengths (sLPXTG, 173 aa; lLPXTG, 431 aa) were used as covalent surface anchors. Expression of B domain in fusion with the LPXTG anchoring domains was confirmed in *L. lactis* cell lysates using SDS-PAGE followed by Coomassie brilliant blue staining (Figure 1A) and Western blotting (Figure 1B). The surface display of the fusion proteins on *L. lactis* was evaluated using flow cytometry (Figure 1C), and was statistically significant in comparison with the empty plasmid-containing control. An increase in MFI was observed for *L. lactis* that displayed B domain with both of the sLPXTG and lLPXTG covalent anchoring domains (using plasmids pSD-sLPXTG and pSD-lLPXTG, respectively; Figure 1). The surface display with the lLPXTG anchor was more effective (2.3-fold the control) than that with the sLPXTG anchor (1.7-fold the control); however, both were significantly lower than that achieved with the non-covalent cAcmA anchor (plasmid pSDBA3b; 8.6-fold the control). The difference in surface display was not due to different expression levels, because the expression level of LPXTG domain fusion was higher than that of cAcmA domain fusion (Figures 1A,B).

### Bioinformatic Identification of Putative Non-covalent Anchoring Domains for *L. lactis*

A total of 13 non-covalent surface anchoring domains, according to Pfam database, were considered in the present study (Supplementary Material S1). Majority of those are more abundant in gram-positive bacteria (phyla Actinobacteria, Firmicutes); however, some are also frequent in gram-negative Proteobacteria (PG_binding_3, SH3_3, PG_binding_1, LysM), while their occurrence in gram-negative Bacteroidetes is low. Anchoring domains SH3_3, PG_binding_1 and PG_binding_4 are the most widespread and each appears in more than 1000 species of bacteria (Supplementary Material S1). The genome of *L. lactis* NZ9000 contains 12 proteins with non-covalent anchoring domains of four different Pfam categories (LysM, CW_1, Cpl-7, WxL; Supplementary Material S1).

Apart from lactococcal proteins, phage endolysins usually require a surface anchoring domain for their activity (Mahony et al., 2013, 2017). Of the 17 lactococcal phage endolysins considered in the present study, only three contain Pfam-categorized non-covalent anchoring domains (of two different types: LysM, SH3_5; Supplementary Material S1). We hypothesized that the rest of the endolysins contain putative anchoring domains of novel types, or that these domains are not recognized by the Pfam search engine due to significant differences in the sequences. To test for the surface display of different anchoring domains, 11 proteins that contain anchoring domains of five different Pfam categories, as well as three putative anchoring domains, were selected for characterization (Table 1). Anchoring domains are often included as multiple repeats (e.g., 3 LysM repeats in the prototype cAcmA anchor); therefore, duplication of short anchors (i.e., CW_1, Cpl-7) was also attempted.

**TABLE 1:** Details of anchoring domains of lactococcal and phage origin used in the present study.

**Organism**	**Accession number**	**Product name**	**PFAM**		**Anchor**		**Presumed target^1^**	**Plasmid**
						
			**Number**	**Domain**	**Amino acids**	**Name**		
*L. lactis* NZ9000	WP_011834544	Hypothetical protein	PF01473	CW_1	69–88	nCW	choline/TA	pSD-CW
	WP_011834816	LysM peptidoglycan – binding domain – containing protein	PF01183	LysM	331–429	c2Lys	PG	pSD-2LysM
	WP_011834547	LysM peptidoglycan – binding domain-containing protein	PF01476	LysM	194–361	cAcmD	PG	pSD-3LysM
	WP_011834353	LysM peptidoglycan – binding domain – containing protein	PF01476	LysM	224–437	cAcmA	PG	pSDBA3b
	WP_011834746	Transglycosylase	PF06737	Cpl-7	32–73	nCPL	PG	pSD-Cpl
	WP_011835003	WxL domain-containing protein	PF13731	WxL	6–242	cWxL1	PG	pSD-WxL1
	WP_011676619	WxL domain – containing protein	PF13731	WxL	31–259	cWxL3	PG	pSD-WxL3
Lactococcal phage sk1	NP_044966	Endolysin			147–246	cSK1		pSD-SK1
Lactococcal phage AM12	ARQ95638	Endolysin			150–344	cAM12		pSD-AM12
Lactococcal phage AM7	ARM66124	Endolysin			137–249	cAM7		pSD-AM7
Lactococcal phage 1358	YP_009140409	Endolysin	PF08460	SH3_5	158–219	c1358	PG	pSD-1358

*^1^According to Desvaux et al. (2018); PG, peptidoglycan; TA, teichoic acid.*

### Constructs for Assessing Surface Display of Anchoring Domains in *L. lactis*

**FIGURE 2:**
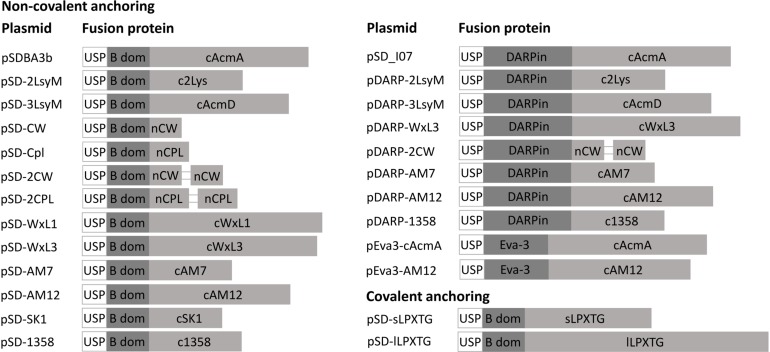
Fusion proteins for the lactococcal surface display. USP, Usp45 signal peptide for secretion to the growth medium (28 aa); B dom, B domain of staphylococcal protein A (58 aa); cAcmA, C-terminal part of AcmA protein-containing 3 LysM repeats (214 aa); c2Lys, anchoring domain containing 2 LysM repeats (118 aa); cAcmD, anchoring domain containing 3 LysM repeats (186 aa); nCW, CW_1 anchoring domain (41 aa); nCPL, CPL-7 anchoring domain (53 aa); cWxL1, cWxL1 anchoring domain (237 aa); cWxL3, cWxL3 anchoring domain (229 aa); cAM7, cAM7 anchoring domain (112 aa); cAM12, cAM12 anchoring domain (192 aa); cSK1, cSK1 anchoring domain (99 aa); c1358, c1358 anchoring domain (126 aa); sLPXTG, sLPXTG anchoring domain (172 aa); lLPXTG, lLPXTG anchoring domain (430 aa); DARPin, DARPin I07 (121 aa); Eva-3; chemokine-binding evasin-3 (85 aa).

The genes for the different lactococcal and phage anchoring domains were cloned into our previously reported plasmid for surface display pSDBA3b, under the control of the NisA promoter (Skrlec et al., 2017). This allowed fusion to the Usp45 secretion signal and reporter proteins (i.e., B domain of staphylococcal protein A, DARPin I07, evasin-3). All of the fusion proteins that were expressed using the plasmids listed in Table 1 are shown schematically in Figure 2.

### Expression of Fusion Proteins for Non-covalent Anchoring on the Surface of *L. lactis*

**FIGURE 3:**
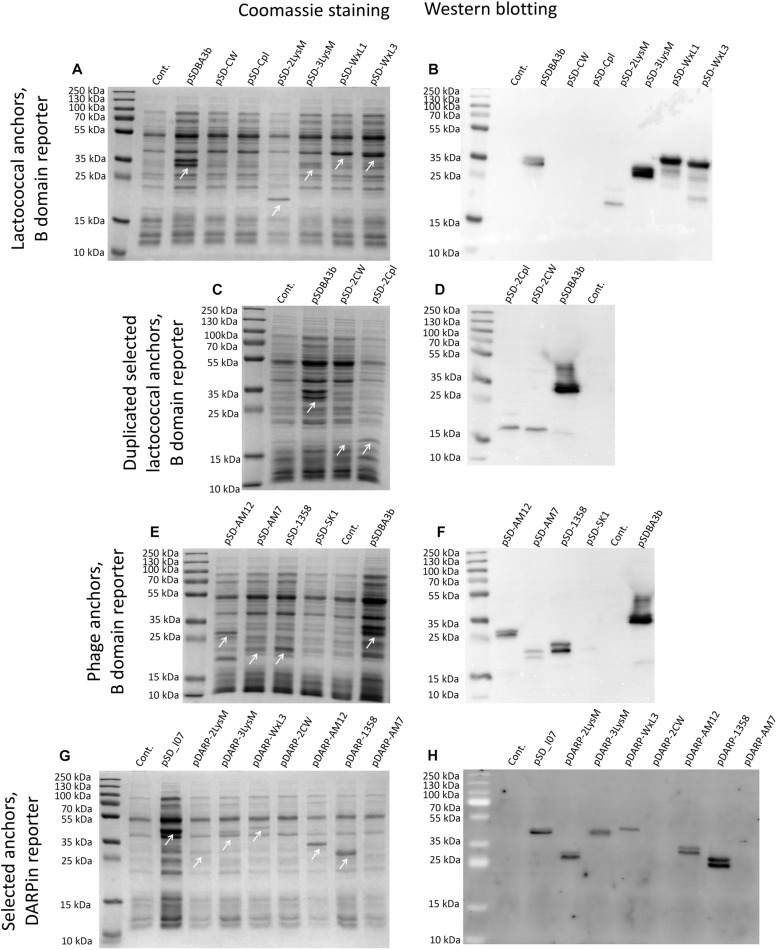
Coomassie blue staining **(A**,**C**,**E**,**G)** and Western blotting **(B**,**D**,**F**,**G)** of lysates of *L. lactis* cells expressing the fusion proteins. B domain in fusion with the Usp45 secretion signal and lactococcal anchoring domains CW, CPL, c2Lys, cAcmD, cWxL1, and cWxL3 **(A**,**B)**. B domain in fusion with the Usp45 secretion signal and lactococcal anchoring domains 2CW and 2CPL **(C**,**D)**. B domain in fusion with the Usp45 secretion signal and phage anchoring domains cAM12, cAM7, c1358, and cSK1 **(E**,**F)**. DARPin I07 in fusion with the Usp45 secretion signal and anchoring domains c2Lys, cAcmD, cWxL3, 2CW, cAM12, c1358, and cAM7 **(G**,**H)**. Cont., control containing empty plasmid pNZ8148. Bands corresponding to the fusion proteins of B domain and anchoring domains **(A**,**C**,**E)**, and of DARPin I07 and anchoring domains **(G)** are indicated with white arrows.

Expression of fusion proteins that consisted of the reporter proteins and the lactococcal or phage surface anchoring domains was assessed in cell lysates using SDS-PAGE, followed by Coomassie brilliant blue staining or Western blotting (Figure 3). Expression of B domain–anchor fusion proteins was observed in the bacterial cultures that contained the following plasmids: pSDBA3b, pSD-2LysM, pSD-3LysM, pSD-WxL1, pSD-WxL3, pSD-AM12, pSD-AM7, and pSD-1358; no expression was seen for the constructs pSD-CW, pSD-Cpl, and pSD-SK1 (Figures 3A,B,E,F). Expression was also detected with the fusion proteins that contained the duplicated CW_1 and Cpl-7 anchors (i.e., pSD-2CW, pSD-2Cpl; Figures 3C,D), possibly due to an increase in molecular weight. Expression of DARPin I07-containing fusion proteins was observed for the following constructs: pSD_I07, pDARP-2LysM, pDARP-3LysM, pDARP-WxL3, pDARP-AM12, and pDARP-1358; no expression was seen for the constructs pDARP-2CW and pDARP-AM7 (Figures 3G,H). Double bands that occurred with anchors cAM12, cAM7 and c1358 were probably the consequence of Usp45 signal sequence removal during the secretion, and presence of two variants of the fusion proteins: one containing Usp45 signal (un-processed; cytoplasmic) and the other without Usp45 signal (processed; cell wall anchored) in the total cell lysate (Dieye et al., 2001; Le Loir et al., 2001). Multiple bands, observed with cAcmA, cWxL1 and cWxL3, were probably the results of both, processing (upper two bands), as well as degradation (lower bands), as observed previously for cAcmA and M6 anchors (Poquet et al., 2000; Dieye et al., 2001).

### Surface Display of Anchoring Proteins in Fusion With B Domain or DARPin I07 on *L. lactis*

**FIGURE 4:**
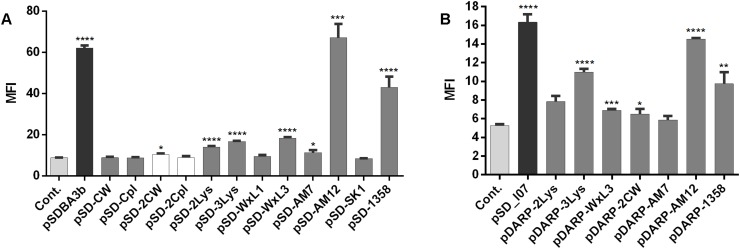
Flow cytometry of *L. lactis* cells expressing the lactococcal/phage anchoring domains in fusion with B domain **(A)** or selected lactococcal/phage anchoring domains in fusion with DARPin I07 **(B)**, detected using an Alexa 488-labeled donkey anti-goat antibody (for B domain fusion proteins), or a FITC-conjugated human IgG (for DARPin I07 fusion proteins). Cont., control containing empty plasmid pNZ8148. MFI, mean fluorescence intensity. Error bars indicate standard deviations. ^∗^*p* < 0.05; ^∗∗^*p* < 0.01; ^∗∗∗^*p* < 0.001; ^∗∗∗∗^*p* < 0.0001 vs. control (Student’s *t*-tests).

The surface display of several fusion proteins was confirmed using flow cytometry (Figure 4). Statistically significant increases in MFI in comparison with the empty plasmid-containing control were seen for B domain-containing fusion proteins encoded by the following plasmids: pSDBA3b, pSD-2LysM, pSD-3LysM, pSD-WxL3, pSD-AM12, pSD-AM7, and pSD-1358 (Figure 4A). The expression with pDARP-2CW could not be detected with western blot (Figure 3H); the weak surface display may therefore be the result of an experimental error. The greatest extent of surface display of B domain was seen for pSD-AM12 (7.5-fold the control), pSDBA3b (sevenfold the control), and pSD-1358 (fivefold the control). Duplicating the CW_1 domain (Figure 4A, plasmid pSD-2CW) led to a small, but significant increase in MFI. No significant surface display was seen using the single CW_1, single Cpl-7, or duplicated Cpl-7 domains.

To confirm the general applicability of surface anchoring domains, B domain was replaced with human Fc binding DARPin I07 in selected fusion proteins (those with significant surface display of B domain), and the surface display was evaluated using flow cytometry (Figure 4B). Statistically significant increases in MFI in comparison with the empty plasmid-containing control were seen for DARPin I07 fusion proteins encoded by the following plasmids: pSD_I07, pDARP-3LysM, pDARP-WxL3, pDARP-2CW, pDARP-AM12, and pDARP-1358. The greatest surface display was again seen with the anchors cAcmA (pSD_I07; threefold the control) and cAM12 (pDARP-AM12; 2.8-fold the control).

Surface display did not correlate with the expression level of fusion proteins, which may be due to the limited efficacy of the Sec secretion pathway that depends on the properties of individual proteins. In *L. lactis*, the secretion efficacy between 30 and 95% was reported for recombinant proteins (Le Loir et al., 2005), indicating that surface display cannot be predicted from the expression level.

### Detection of B Domain – cAcmA and B Domain – cAM12 Fusion Proteins After Subcellular Fractionation

**FIGURE 5:**
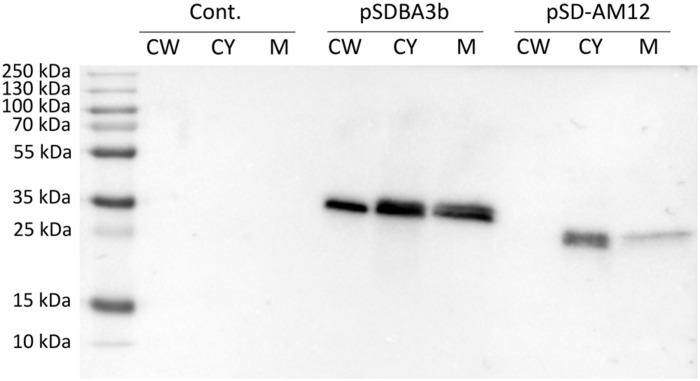
Western blotting of fractionated cell lysates of *L. lactis* cells expressing fusion of Usp45 secretion signal, B domain and, either lactococcal anchoring domain cAcmA (plasmid pSDBA3b), or phage anchoring domain cAM12 (plasmid pSD-AM12). Cell wall (CW), cytoplasmic (CY), and membrane (M) fractions of the cell lysates were analyzed. Cont., control containing empty plasmid pNZ8148.

Presence of B domain–cAcmA and B domain–cAM12 fusion proteins in cell wall, cytoplasmic and membrane fractions was assessed using SDS-PAGE and Western blot. B domain–cAcmA fusion protein was detected in all three fractions, while B domain–cAM12 was detected in cytoplasmic and membrane fraction, but not in the cell wall fraction (Figure 5).

### Heterologous Coating of Non-recombinant *L. lactis* With B Domain – cAcmA and B Domain – cAM12 Fusion Proteins

**FIGURE 6:**
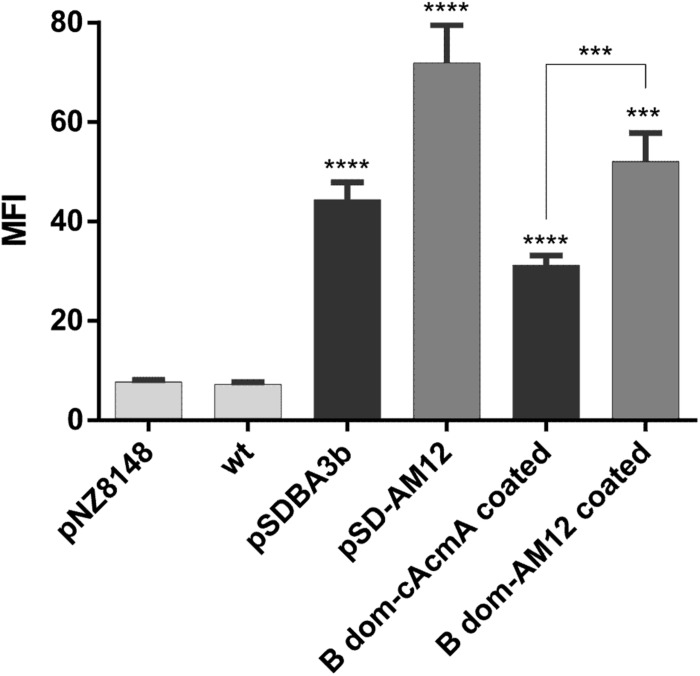
Flow cytometry of *L. lactis* cells coated with B domain-cAcmA or B domain-cAM12 fusion proteins from the conditioned media of their corresponding recombinant cultures that contained plasmids pSDBA3b and pSD-AM12, respectively. pSDBA3b- and pSD-AM12-containing *L. lactis* served as the positive controls. *L. lactis* that contained the empty plasmid pNZ8148 and wild-type *L. lacti*s (wt) served as the negative controls. B domain was detected with a goat anti-protein A antibody, and DARPin I07 with an Alexa 488 labeled donkey anti-goat antibody. MFI, mean fluorescence intensity. Error bars indicate standard deviations. ^∗∗∗^*p* < 0.001; ^∗∗∗∗^*p* < 0.0001 vs. control, and as indicated (Student’s *t*-tests).

Non-recombinant *L. lactis* cells were incubated in conditioned media of recombinant *L. lactis* that contained either the B domain–cAcmA fusion protein (encoded by plasmid pSDBA3b) or the B domain–cAM12 fusion protein (encoded by plasmid pSD-AM12). Significant heterologous surface display was seen for both of these when assessed as MFI by flow cytometry, as B domain–cAcmA-coated non-recombinant *L. lactis* (fourfold the control), and B domain–cAM12-coated non-recombinant *L. lactis* (sevenfold the control) (Figure 6). The extent of heterologous surface display was lower than that achieved with recombinant *L. lactis* cells that contained pSDBA3b or pSD-AM12 (positive controls; MFI: 5.7-fold and ninefold the control, respectively). However, the cAM12 anchor promoted greater surface display with both of these reporter proteins (B domain, DARPin I07).

### Comparison of cAcmA and cAM12 Surface Anchors for the Surface Display of CXCL8-Binding Evasin-3 on *L. lactis*

**FIGURE 7:**
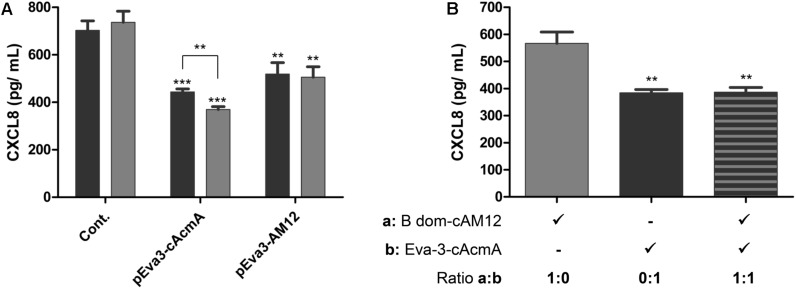
ELISA-determined concentrations of CXCL8 after incubations with two concentrations of *L. lactis* cells (2 × 10^9^ cells/mL, black bars; 6 × 10^9^ cells/mL, gray bars) that displayed evasin-3 with the cAcmA anchor (pEva3-cAcmA) or the cAM12 anchor (pEva3-AM12) **(A)**, or after incubation with non-recombinant *L. lactis* cells (2 × 10^9^ cells/mL) coated with either evasin-3–cAcmA fusion protein, B domain–cAM12 fusion protein, or equal amounts of both fusion proteins **(B)**. Cont., control containing empty plasmid pNZ8148. Experiments were carried out in triplicate. Error bars indicate standard deviations. ^∗∗^*p* < 0.01; ^∗∗∗^*p* < 0.001; vs. control, and as indicated (Student’s *t*-tests).

The cAcmA and cAM12 surface anchors were compared for their promotion of surface display on *L. lactis* of the chemokine CXCL8-binder, evasin-3, which was previously proposed as a relevant therapeutic protein (Skrlec et al., 2017). As determined using ELISA, both of these recombinant *L. lactis* species bound to, and thus removed, human chemokine CXCL8 from solution. The extent of removal of CXCL8 was a little greater (38%) with *L. lactis* with evasin-3 via the cAcmA anchor (2 × 10^9^ cells/mL) than that achieved with the same concentration of *L. lactis* with evasin-3 via the cAM12 anchor (28%). Higher concentrations of evasin-3–displaying *L. lactis* cells (6 × 10^9^ cells/mL) removed a larger portion of CXCL8 when using the cAcmA anchor (49%). However, when using the cAM12 anchor, increased cell concentrations to 6 × 10^9^ cells/mL had no significant effects on CXCL-8 removal (30%; Figure 7A); this may be due to different anchoring mode in comparison to cAcmA. To assess the possible competition between cAcmA and cAM12 surface anchors, non-recombinant *L. lactis* coated with evasin-3-cAcmA and B domain–3–cAM12 fusion proteins were analyzed for CXCL8 binding. No significant difference in the extent of removal of CXCL8 was observed between evasin-3-cAcmA-coated *L. lactis* and *L. lactis* coated with equal amounts of evasin-3-cAcmA and B domain-cAM12 fusion proteins (Figure 7B). This suggests that concomitant coating with both, cAcmA- and cAM12-containing fusion proteins did not impair cAcmA-mediated coating.

## Discussion

In line with previous successful applications of LPXTG domains [e.g., proteins PrtP (Ramasamy et al., 2006; Liu et al., 2011), M6 (Piard et al., 1997; Dieye et al., 2001)] for lactococcal surface display, in the present study, another two LPXTG domains were introduced for covalent anchoring on the lactococcal surface. Both of these are C-terminal parts of aggregation-associated sex factor CluA (Godon et al., 1994; Stentz et al., 2004), but of different lengths (sLPXTG, 173 aa; lLPXTG, 431 aa). Both of these anchors were successfully used for surface display of the model protein B domain, with this surface display higher with the longer variant, lLPXTG. This is in agreement with previous studies, where the larger of two PrtP anchors provided better anchoring (Davis and Model, 1985; Lindholm et al., 2004; Kyla-Nikkila et al., 2010). Unfortunately, the extent of surface display achieved with these LPXTG domains was considerably lower than that achieved with the non-covalent cAcmA domain, which has been the most often used for surface display on *L. lactis* (Okano et al., 2008; Zadravec et al., 2015b; Jee et al., 2017; Skrlec et al., 2017). Our results here are similar to those obtained with the PrtP-derived LPXTG anchor, which was also not as good as the cAcmA anchor (Lindholm et al., 2004). The difference in surface display might be due to the positioning of the LPXTG–peptidoglycan bond deep in the peptidoglycan lattice close to the membrane, and consequently to its lower exposure to the environment in comparison to surface attached cAcmA domain. Moreover, LPXTG binding is catalyzed by the enzyme sortase A, and its availability might be a limiting factor for this surface display. We therefore focused most of our efforts on the identification of new non-covalent anchors that would theoretically allow for their removal from the membrane, and provide better exposure in the upper layers of the peptidoglycan.

Using a bioinformatics approach, 12 proteins with non-covalent anchoring domains were identified in the genome of *L. lactis*, which included our reference anchor cAcmA. cAcmA contains three LysM repeats, and its binding to the surface has been characterized in detail (Steen et al., 2005; Buist et al., 2008). The non-covalent anchors belong to four different Pfam categories: LysM, CW_1, Cpl-7, and WxL. LysM repeats are included in the previously mentioned anchor cAcmA as well as in AcmD (Visweswaran et al., 2013). CW_1, Cpl-7, and WxL, on the other hand, have not been applied as anchors in *L. lactis* to date, to the best of our knowledge. In the present study we also focused on 17 lactococcal phages’ endolysins. Although according to the Pfam analysis all of the endolysins contained an N-terminal hydrolytic domain, only two different C-terminal cell-wall binding domains were identified (i.e., LysM and SH3); several endolysins contained no apparent cell-wall binding domain. This suggested the possibility of new putative anchoring domains.

The surface display that was achieved with these different anchors was benchmarked against the well-established cAcmA anchor. All of the lactococcal non-covalent anchors were not as good as cAcmA, although significant surface display was seen with the cAcmD (Pfam: LysM) and cWxL3 (Pfam: WxL) anchors, with both of the reporter proteins. Apart from lactic acid bacteria (genera *Lactococcus* and Lactobacillus), LysM has also been applied in *Staphylococcus aureus, Bacillus thuringensis* and *Bacillus subtilis* (Visweswaran et al., 2014). The use of the WxL domain for surface anchoring was reported previously for *Enterococcus faecalis* (Brinster et al., 2007). Degradation of LysM and WxL domains that has been observed should be taken into account in practical applications, particularly regarding the temporal stability of the displayed proteins. The nCW anchor (Pfam: CW_1) is a choline-binding domain, which binds to teichoic acid and lipoteichoic acid (Fernandez-Tornero et al., 2001), but has not been applied for recombinant surface display yet. In our study, it promoted significant surface display, although only when two repeats were introduced. This is in agreement with the CW_1 domain organization in CW_1-containing proteins, as these can, according to the Pfam database, contain numerous repeats of CW_1 (i.e., up to 60). Inclusion of multiple repeats also suggests a possible direction for further studies of this surface display on *L. lactis*.

Two lactococcal non-covalent anchors of phage origin (cAM12, Pfam: unrecognized; c1358, Pfam: SH3) promoted high levels of surface display of both of the reporter proteins; none of them, according to our knowledge, has been previously applied for recombinant surface display. Among all of the new anchors tested in the present study, cAM12 promoted the highest surface display, which was comparable to that achieved with the benchmark anchor cAcmA. No Pfam domains were recognized in cAM12 in Pfam searches; however, using manual alignment, part of the protein was annotated as clostridial hydrophobic with a conserved W (ChW) domain (Pfam number: PF07538; Supplementary Figure S1). ChW domains were first identified in the genus *Clostridium* (Nolling et al., 2001), but according to the Pfam database, they have since been observed in more than 200 species, including the genera *Lactococcus* and *Lactobacillus.* cAM12 probably attaches to a different cell surface moiety than cAcmA, as the former was not detected in the cell wall fraction obtained by the lysozyme/mutanolysin degradation of the peptidoglycan. This may suggest that cAM12 attaches to the cell membrane, which would be surprising due to the significant exposure of fusion protein achieved with cAM12. Alternatively, cAM12 may attach to the non-peptidoglycan membrane-anchored component of the cell wall, such as lipoteichoic acid (Desvaux et al., 2018).

Among all of the anchors tested in the present study, the cAM12 anchor can be regarded as the most promising new non-covalent anchor for lactococcal surface display. cAM12 was therefore assessed in two applications with potential therapeutic relevance. In the first, we used heterologous coating of wild-type *L. lactis* with fusion proteins that contained the anchoring domain and reporter protein (B domain). This approach was previously described as a potential “non-GMO” approach to surface display (Zadravec et al., 2015a). The cAM12 anchor enabled higher heterologous surface display, compared with the cAcmA anchor. In the second approach, we used the cAM12 anchor to attach evasin-3 to the lactococcal surface, and *L. lactis* with surface display of evasin-3 was shown to decrease the production of CXCL8 in the Caco-2 cell line (Skrlec et al., 2017). This bacterium was therefore suggested as a possible treatment for inflammatory bowel disease. Here, evasin-3–displaying bacteria that used the cAM12 anchor were less effective for the removal of CXCL8 than the corresponding bacteria with the cAcmA anchor; however, significant removal of CXCL8 was achieved with both of these anchors. Moreover, heterologous attachment of evasin-3 to *L. lactis* via cAcmA anchoring domain, and subsequent removal of CXCL8, was not diminished by concomitant addition of mock cAM12-containing fusion protein, substantiating the observation that cAM12 and cAcmA bind to different surface moieties and could be used complementarily.

To conclude, although LPXTG anchors can display model proteins, they were inferior to the LysM-repeat-containing C-terminus of AcmA. Lactococcal and phage non-covalent anchors were therefore systematically screened in the present study for promotion of surface display of heterologous proteins on *L. lactis*, and several new anchors were identified. The C-terminal part of phage AM12 endolysin (cAM12) was shown to contain a ChW domain that promotes high surface display, comparable to that of the C-terminus of AcmA. cAM12 promoted the surface display of several reporter proteins (i.e., B domain, DARPin I07, and evasin-3), and it therefore represents a useful alternative for surface display on *L. lactis*.

## Data Availability

The raw data supporting the conclusions of this manuscript will be made available by the authors, without undue reservation, to any qualified researcher.

## Author Contributions

All authors conceived the study, designed the experiments, contributed to the manuscript revision, and read and approved the submitted version. TP performed the laboratory work and analyzed the data. TP and AB wrote the manuscript.

## Conflict of Interest Statement

The authors declare that the research was conducted in the absence of any commercial or financial relationships that could be construed as a potential conflict of interest.

## Footnotes

1 https://pfam.xfam.org/

2 https://img.jgi.doe.gov/
