# Supplementary material for: Screening for New Surface Anchoring Domains for Lactococcus lactis
Source: Front Microbiol. 2019 Aug 13;10:1879. doi: 10.3389/fmicb.2019.01879 (PMC6700490; doi:10.3389/fmicb.2019.01879)
Supplement: Supplementary file 1 [file Data_Sheet_1.docx]

Supplementary material

Screening for new surface anchoring domains for *Lactococcus lactis*

Tina Vida Plavec^1,2^, Borut Štrukelj^1,2^, Aleš Berlec^1,2*^

^1^Department of Biotechnology, Jožef Stefan Institute, Jamova 39, Ljubljana, Slovenia

^2^Faculty of Pharmacy, University of Ljubljana, Aškerčeva 7, Ljubljana, Slovenia

*** Correspondence:**Aleš Berlec
ales.berlec@ijs.si

**Table S1.** Strains, primers, plasmids and synthetic genes used in this study.

| **Strain, primer plasmid, or gene** | **Relevant features or sequence** | **Reference** |
| --- | --- | --- |
| **Strains** |  |  |
| *E. coli* DH5α | endA1 glnV44 thi-1 recA1 relA1 gyrA96 deoR F^-^ Φ80d*lacZ*ΔM15 Δ(*lacZYA-argF*)U169, hsdR17(r_K_^-^ m_K_^+^), λ– | Invitrogen |
| *L. lactis* NZ9000 | MG1363 *nisRK* Δ*pepN* | NIZO |
| **Primers** |  |  |
| 2xLysM-F-Eco | 5’- GAATTCTCCGTTCATGATGTGGAAAC-3’ | This work |
| 2xLysM-R-TAA-Xba | 5’- TCTAGATTAATAATTTAAAGTTTGACCAGCATAAATC-3’ | This work |
| 3xLysM-F-Eco | 5’-GAATTCTCTAGTGCGAGCGCTGTTAC-3’ | This work |
| 3xLysM-R-TAA-Xba | 5’- TCTAGATTAAATTTTAATGGTTTGGCCTGG-3’ | This work |
| CW_1-F-Eco | 5’- GAATTCTCAACGATTAAAGTGGTTAATAAATC-3’ | This work |
| CW_1-R-Spe | 5’- TTAATAACTAGTCATGACACCCGTTGATGAATC-3’ | This work |
| CW_1-R-TAA-Xba | 5’- TCTAGATTACATGACACCCGTTGATGAATC-3’ | This work |
| Cpl-7-F-Eco | 5’- GAATTCGGCGGAATCGCTCATGCG-3’ | This work |
| Cpl-7-R-Spe | 5’- TTAATTACTAGTTGTCAGAATGTTATTAACTTCTGTTTG-3’ | This work |
| Cpl-7-R-TAA-Xba | 5’-TCTAGATTATGTCAGAATGTTATTAACTTCTGTTTG-3’ | This work |
| WxL1-F-Eco | 5’-GAATTCGCGACAGTAGGAAATACATCAAG-3’ | This work |
| WxL1-R-TAA-Xba | 5’-TCTAGATTAACTATAAAGATTCCAGTTTAAAGTTGTAG-3’ | This work |
| WxL3-F-Eco | 5’-GAATTCACTGGGGGAGATTATACATC-3’ | This work |
| WxL3-R-TAA-Xba | 5’-TCTAGATTAATTCGTTAAGGACCAAATTAATTGTG-3’ | This work |
| AM7-F-Eco | 5’-AGAATTCAGTTGGGCTCTTCATGGAC-3’ | This work |
| AM7-R-Xba | 5’-TTCTAGATTATGGTTTAGTAGCCGTTACTCC-3’ | This work |
| AM12-F-Eco | 5’-TGAATTCTATTTAGCTCGCATTGGG-3’ | This work |
| AM12-R-Xba | 5’-TTCTAGATTAAAATGGATTTGTATCACTAATTGC-3’ | This work |
| SK1-F-Eco | 5’-TGAATTCTATTTCATCACGGAGCTTAATAAAC-3’ | This work |
| SK1-R-Xba | 5’-TTCTAGATTATTTTTTAGCAATGATAGGCTTATC-3’ | This work |
| 1358-F-Eco | 5’-AGAATTCATGGTTACTGGTAAAGGAC-3’ | This work |
| 1358-R-Xba | 5’-TTCTAGATTAATAACAAAGACCTGTTCGG-3’ | This work |
| Clu1072-F-Eco | 5’-AGAATTCGGAAATAAATCATTGATGACAAATG-3’ | This work |
| Clu813-F-Eco | 5’-AGAATTCGTCCCTCCAAAAGTAGTG-3’ | This work |
| Clu-R-Xba | 5’-TTCTAGATTATTTACGAATCTTACGAACAG-3’ | This work |
| **Plasmids** |  |  |
| pGEM-T Easy | Ap^r^, cloning vector for PCR products | Promega |
| pNZ8148 | pSH71 derivative, P*_nisA_,* Cm^r^, nisin-controlled expression | (Kuipers et al., 1993; de Ruyter et al., 1996; Mierau and Kleerebezem, 2005) |
| pSDBA3b | pNZ8148 containing gene fusion of *sp*_Usp45,_ *b-dom* and *acmA3b* | (Skrlec et al., 2017) |
| pSD-2LysM | pNZ8148 containing gene fusion of *sp*_Usp45_, *b-dom* and *2lysm* | This work |
| pSD-3LysM | pNZ8148 containing gene fusion of *sp*_Usp45_, *b-dom* and *3lysm* | This work |
| pSD-CW | pNZ8148 containing gene fusion of *sp*_Usp45_, *b-dom* and *cw* | This work |
| pSD-Cpl | pNZ8148 containing gene fusion of *sp*_Usp45_, *b-dom* and *cpl* | This work |
| pSD-2CW | pNZ8148 containing gene fusion of *sp*_Usp45_, *b-dom* and *2cw* | This work |
| pSD-2Cpl | pNZ8148 containing gene fusion of *sp*_Usp45_, *b-dom* and *2cpl* | This work |
| pSD-WxL1 | pNZ8148 containing gene fusion of *sp*_Usp45_, *b-dom* and *wxl1* | This work |
| pSD-WxL3 | pNZ8148 containing gene fusion of *sp*_Usp45_, *b-dom* and *wxl3* | This work |
| pSD-AM7 | pNZ8148 containing gene fusion of *sp*_Usp45_, *b-dom* and *am7* | This work |
| pSD-AM12 | pNZ8148 containing gene fusion of *sp*_Usp45_, *b-dom* and *am12* | This work |
| pSD-SK1 | pNZ8148 containing gene fusion of *sp*_Usp45_, *b-dom* and *sk1* | This work |
| pSD-1358 | pNZ8148 containing gene fusion of *sp*_Usp45_, *b-dom* and *1358* | This work |
| pSD_I07 | pNZ8148 containing gene fusion of *sp*_Usp45_, *darpin* and *acmA3b* | This work |
| pDARP-2LysM | pNZ8148 containing gene fusion of *sp*_Usp45_, *darpin* and *2lysm* | This work |
| pDARP-3LysM | pNZ8148 containing gene fusion of *sp*_Usp45_, *darpin* and *3lysm* | This work |
| pDARP-2CW | pNZ8148 containing gene fusion of *sp*_Usp45_, *darpin* and *2cw* | This work |
| pDARP-WxL3 | pNZ8148 containing gene fusion of *sp*_Usp45_, *darpin* and *wxl3* | This work |
| pDARP-AM7 | pNZ8148 containing gene fusion of *sp*_Usp45_, *darpin* and *am7* | This work |
| pDARP-AM12 | pNZ8148 containing gene fusion of *sp*_Usp45_, *darpin* and *am12* | This work |
| pDARP-1358 | pNZ8148 containing gene fusion of *sp*_Usp45_, *darpin* and *1358* | This work |
| pSD-sLPXTG | pNZ8148 containing gene fusion of *sp*_Usp45_, *b-dom* and *slpxtg* | This work |
| pSD-lLPXTG | pNZ8148 containing gene fusion of *sp*_Usp45_, *b-dom* and *llpxtg* | This work |
| pEva3-cAcmA | pNZ8148 containing gene fusion of *sp*_Usp45_, *eva3* and *acmA3b* | (Skrlec et al., 2017) |
| pEva3-AM12 | pNZ8148 containing gene fusion of *sp*_Usp45_, *eva3* and *am12* | This work |
| **Synthetic genes** |  |  |
| *am7* | AGTTGGGCTCTTCATGGACAATCAGTTCAGGCAGTTCAAGACTATTTTATTGCTGGTATCCAACGATACATGGGTGGGGCATCTACTTCAACGGGTAATGATTATACATCTCAAAATACAACATATACGTTGAATAAGGAATTACTTGATATGTATCTTATATTTACAGTTGACACGAAACGTTGGTATATTTCAAATGGCGTTGGTGTTCGTTATGTCCGTACAACTAGAATGCTTGCAAATTATCAAGATAATTTCGGTAAACTTCAATTACCTACAGACAAAATGTATCAAGTTGAATTAGATAAAGAATTTGGAGTAACGGCTACTAAACCATAA | This work |
| *am12* | TATTTAGCTCGCATTGGGATTTCTAAAAATCAATTGGCAAATGACCTTGCGCATGGAGTAGGCGGTACAGTTCCAGCCCCAACATCAAAGCCAGCACCAGCTCCAGCTTCAAAACCTAGTAAATCAGCTCCAAAGGTACAAACAAATGTTGTTTATGGTTTACATCAAAAAGGGGGAGGGTGGTTAGGTGAAATTACGAATTTCAATAATAGTAATAGTTCAGGATTTGCTGGTTTGCCAAGTAATTCACATGATTTGTTATATATGCGTGTTACACACGGAGCTTTAAAATACCGTGTTCATACAATTGAAGATGGTTGGTTGGGTTGGGTTACTAACGGAAATAAAAACGATACCGTAAATGGCTGCGCAGGTATTTCTGGTCATACTATTGATGGGGTACAAGCATATTTTTACACTCCAAGTGGCGAAGTCTATCAACAGGCCCACTATCGTTCACAAACGGCCAAACGTGCTGGATGGTTAGCTCCAGTTGTAGATGATTCTGATTTTGCTGGCATTTTTGGTGAACCATTAGACCGTTTGCAAGTAGCAATTAGTGATACAAATCCATTTTAA | This work |
| *sk1* | TATTTCATCACGGAGCTTAATAAACGCCTTACAGGACAAAACAACACACAAACTAATACTGAGTTGGAAGATGATGATTTAATGAAGTTTACATATACCAACGGAGATAAAACCACATATTATTTTAACGGCGAGAAAGTTATTGCTTTGTCTCATCCAGATCAATTGGCTATTGTGCGTAAAACATATAAAGAGACTACAGGAAAGGATTTGAAGAATTTTGATTGGAAAGGTTCTCCAATTGATATAAGATTCATGCAAGCAAACGGTATTGATAAGCCTATCATTGCTAAAAAATAA | This work |
| *1358* | ATGGTTACTGGTAAAGGACAAGCAGCAGATAAGGCAGCCATTAACACTCGTCCTTATCCAAGTGAATTTTGGGGAGTTGTACGCCCACCTATTACATCAGGAGGGAATATTACTCCTCCTGACCAAAACGGCACTGGAGGGAAACTTACAGCTCAAAGAGGTACATTTAAAGCCAACTCTGCTGTAAATATAAGACGTGCTCCTAATACCAAAACAGGCACAGTGGCTGGAGTACTTAAAGCTGGTCAAACAGTCAACTATGATAATATCATTGACGCAGATGGATGGCGTTGGGTTTCTTGGGTTGGCGCTTCTGGTAATCGAAATTATTCTGCTGTACGACGCTTATCTGATAATTTCCGAACAGGTCTTTGTTATTAA | This work |


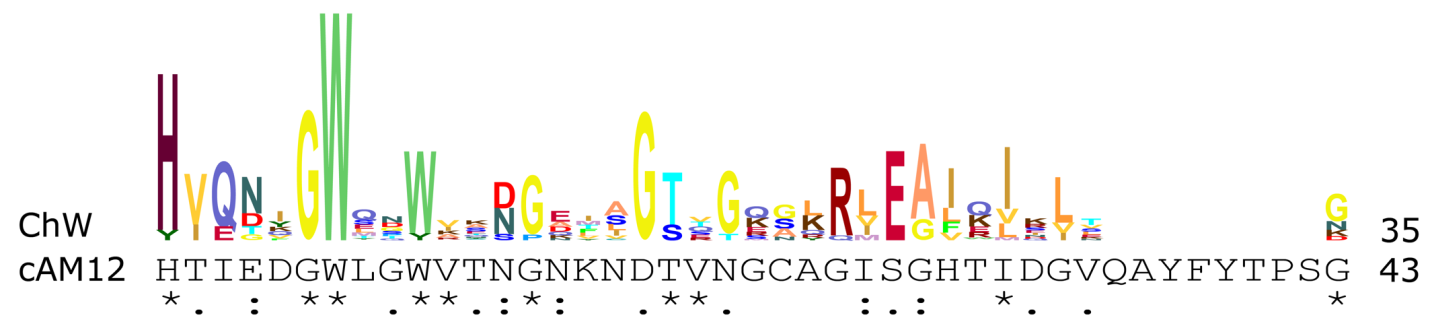


**Figure S1.** Clustal W alignment of the conserved ChW sequence (HMM logo from Pfam; upper row) with part of the cAM12 sequence (middle row). *, conserved residues; colon (:), similar residues; dot (.), residues with weak similarity.

**References**

de Ruyter, P.G., Kuipers, O.P., and de Vos, W.M. (1996). Controlled gene expression systems for *Lactococcus lactis* with the food-grade inducer nisin. *Appl. Environ. Microbiol.* 62(10)**,** 3662-3667.

Kuipers, O.P., Beerthuyzen, M.M., Siezen, R.J., and De Vos, W.M. (1993). Characterization of the nisin gene cluster nisABTCIPR of *Lactococcus lactis*. Requirement of expression of the nisA and nisI genes for development of immunity. *Eur. J. Biochem.* 216(1)**,** 281-291. doi: 10.1111/j.1432-1033.1993.tb18143.x

Mierau, I., and Kleerebezem, M. (2005). 10 years of the nisin-controlled gene expression system (NICE) in *Lactococcus lactis*. *Appl. Microbiol. Biotechnol.* 68(6)**,** 705-717. doi: 10.1007/s00253-005-0107-6

Skrlec, K., Pucer Janez, A., Rogelj, B., Strukelj, B., and Berlec, A. (2017). Evasin-displaying lactic acid bacteria bind different chemokines and neutralize CXCL8 production in Caco-2 cells. *Microb. Biotechnol.* 10(6)**,** 1732-1743. doi: 10.1111/1751-7915.12781
